# Co-culture with neonatal cardiomyocytes enhances the proliferation of iPSC-derived cardiomyocytes via FAK/JNK signaling

**DOI:** 10.1186/s12861-016-0112-2

**Published:** 2016-05-04

**Authors:** Dongbo Ou, Qi Wang, Yanjin Huang, Di Zeng, Ting Wei, Lu Ding, Xiaoli Li, Qiangsun Zheng, Yan Jin

**Affiliations:** State Key Laboratory of Military Stomatology, Research and Development Center for Tissue Engineering, School of Stomatology, Fourth Military Medical University, 1st Kang-fu Road, Xi’an, 710032 Shaanxi China; Department of Cardiology, NO. 422 Hospital of PLA, Zhanjiang, 524005 Guangdong China; Department of Cardiology, Tangdu Hospital, Fourth Military Medical University, Xi’an, 710038 Shaanxi China; Research and Development Center for Tissue Engineering, Fourth Military Medical University, 1st Kang-fu Road, Xi’an, 710032 Shaanxi China

**Keywords:** Induced pluripotent stem cells, Cardiomyocytes, FAK/JNK, Co-culture, Proliferation

## Abstract

**Background:**

We previously reported that the pluripotent stem cells can differentiate into cardiomyocytes (CMs) by co-culture with neonatal CMs (NCMs) in vitro. However, the involving mechanism is not clear.

**Methods:**

Mouse induced pluripotent stem cells (iPSCs) were cultured in hanging drops to form embryoid bodies (EBs) and to induce myocardial differentiation. Co-culture of EBs and NCMs was established in a transwell insert system, while EBs grown alone in the wells were used as controls.

**Results:**

Co-culture with NCMs markedly increased the generation of functional CMs from iPSCs. The focal adhesion kinase (FAK) phosphorylation, and c-Jun N-terminal kinase (JNK) phosphorylation in co-culture were higher than that in EBs grown alone. Treating FAK small interfering RNA (FAK siRNA) or specific inhibitor for JNK (SP600125) to iPSCs significantly reduced the phosphorylation of JNK and the expressions of Mef2c and Bcl-2. The expressions of cTnT and MLC-2V were also decreased. Our results revealed that co-culture with NCMs significantly enhance the differentiation ability of iPSCs by increasing Mef2c and Bcl-2 expressions concomitantly with a marked augment on cell proliferation through JNK signaling pathways.

**Conclusions:**

These findings indicated that co-culture of EBs with NCMs induces genes expressed in a mature pattern and stimulates the proliferation of iPSC-derived CMs (iPS-CMs) by activating FAK/JNK signaling.

**Electronic supplementary material:**

The online version of this article (doi:10.1186/s12861-016-0112-2) contains supplementary material, which is available to authorized users.

## Background

Induced pluripotent stem cells (iPSCs) can differentiate into cardiomyocytes (CMs) with cardiac-specific molecular, structural, and functional properties [1, 2]. iPSC-derived CMs (iPS-CMs) have ionic currents and channel gating properties that are quantitatively similar to those reported for human cardiac myocytes [3]. These iPS-CMs are an important source of CMs for regenerating injured myocardium. However, the successful use of iPS-CMs in cardiac tissue engineering requires an understanding of the important scaffold properties and culture conditions to promote cell attachment, differentiation, organization, and contractile function.

Previously, we had investigated the effect of in vitro cardiac microenvironment on the development and differentiation of embryoid bodies (EBs) and had established a novel embryonic stem cell (ESC) differentiation model that can reproduce the process of cardiovascular development and promote ESC differentiation [4]. However, the involving mechanism is not clear. Interestingly, we found that the expression of focal adhesion kinase (FAK) is induced concurrently with the emergence of cardiovascular progenitor cells during myocardial differentiation. The FAK is a tyrosine kinase that is activated in response to integrin-matrix interactions, which plays a central role in the regulation of cell proliferation, survival, differentiation, and migration [5]. Tyr^397^ is a major autophosphorylation site in the central catalytic domain of FAK. Integrin-matrix interaction signalling at the cell membrane leads to phosphorylation of FAK at Tyr^397^ with subsequent binding of Src and second-step phosphorylation of FAK at Y576/577 [6]. It is reported that the FAK is a key regulator of cardiogenesis in mouse ESCs [7], but the detailed mechanism that the FAK involves in induce myocardial differentiation in pluripotent stem cells is still unclear. c-Jun N-terminal kinase (JNK), plays a critical role in the induction of gene transcription, has been reported to be the downstream effector of FAK [8]. It is reported that JNK plays a key role in the differentiation of ESCs [9]. However, the involving mechanism of FAK/JNK signaling in the development of pluripotent stem cells is not clear. Here, based on previous work, we sought to determine the mechanism of local microenvironments and FAK/JNK that influences the development of iPSCs.

## Methods

### The culture of iPSCs

Mouse iPSCs (miPSCs), carrying a transgenic Oct4-GFP^+^ promoter, were kindly provided by Prof. Duanqing Pei (Chinese Academy of Sciences) [10]. Undifferentiated miPSCs were cultured on a mitotically inactivated mouse embryonic fibroblast (MEF; 50,000 cells/cm^2^) feeder layer as described previously [11]. The culture medium consisted of 85 % knockout high-glucose DMEM with sodium pyruvate, supplemented with 15 % serum replacement, 2 mM GlutaMAX, 0.1 mM nonessential amino acid stock, 0.1 mM β-mercaptoethanol (all from Invitrogen, Carlsbad, CA), and 1000 U/ml murine leukemia inhibitory factor (Chemicon, Temecula, CA). The culture medium was changed daily, and the cells were passaged every 2–3 days to maintain their undifferentiated state.

Before the initiation of EB formation and differentiation, the iPSC colonies were passaged up to 3 times on gelatin-coated dishes without feeder cells to eliminate contaminating MEFs. Before EB induction, adherent cells were enzymatically dissociated into single cells using 0.05 % Trypsin-EDTA (Invitrogen). Then the iPSCs were cultured in hanging drops (400 cells per 20 μl) for 2 days to form uniform-sized EBs.

After pipetting the drops (20 μL) onto lids, lids were placed back on 6- or 12- well plates containing 0.5 ~ 1 mL/well of differentiation medium to prevent drying out of hanging drops. Each time there were 50 EBs formed in a 6- or 12- well plate. Each EB is basically contain the same number of cells before their differentiation. On Day 3 of a hanging drop culture, the cell aggregates were transferred and cultured in suspension in cell culture flasks (BD Bioscience) with differentiation medium for 2–3 days. 5- or 6-day-old EBs were carefully transferred to 6- or 12- well plates coated with 0.1 % gelatin and continuously cultured in differentiation medium. The differentiation medium was based on high-glucose DMEM and supplemented with 20 % ES-qualified fetal bovine serum (Gibco), 2 mM GlutaMAX, 0.1 mM nonessential amino acids, and 0.1 mM β-mercaptoethanol.

### Indirect co-culture model

For co-culture, neonatal CMs (NCMs) were obtained from neonate mouse ventricle as our previous description [4]. Animal experiments were approved by the Laboratory Animal Ethics Committee of the Fourth Military Medical University and were conducted in accordance with national guidelines for the care and use of laboratory animals. Myocyte isolation was conducted in accordance to Institutional Animal Care and Use Committee Guidelines. The NCMs were isolated from 1-day-old αMHC-GFP transgenic mice (from our laboratory). The CMs, which expressed GFP, were sorted from the mixed cells by reporter-based fluorescence-activated cell sorting (FACS) as previous description [4]. The sorted NCMs were co-cultured with EBs in DMEM supplemented with 20 % ES cell-qualified FBS (Gibco), 2 mM GlutaMAX (Invitrogen), 0.1 mM nonessential amino acid (Invitrogen) at a density of 2 × 10^4^ ells/cm^2^. Mouse embryo fibroblasts (MEFs), a very common cell line, were obtained from our laboratory.

The Millicell™ hanging cell culture inserts (PET membranes with 1 μm pores) (Millipore, Bedford, MA, USA) are especially used for co-culture. 5- or 6-day-old EBs were transferred to the 6- or 12- well plate coated with 0.1 % gelatin, then the inserts were placed in some well of the 6- or 12- well plate followed by NCMs or MEFs seeding on the upper compartment (insert) to stay physically separated from the subnatant EBs. Culture medium was the differentiation medium and changed every day.

### MTT assay

Briefly, iPSCs at a concentration of 2 × 10^4^ cells/cm^2^ were cultured for 1–16 days in 12-well plates, 100 μl of MTT solution (5 mg/ml in PBS) was added to each well, and the cells were incubated at 37 °C in a humidified atmosphere of 5 % CO2 for 4 h. Then, the medium was aspirated, and 500 μL of dimethyl sulfoxide was added to dissolve the blue crystals that formed in the cells. After being jolted by the shaker for 15 min, 100 μl of the solution was transferred to a 96-well plate. The optical density (OD) values were determined with a Bio-Rad Model 680 microplate reader at a wavelength of 570 nm. Three replicates were read for each sample, the mean value of 3 was used for final result.

### Semi-quantitative reverse transcription-PCR

Total RNA was extracted using an RNeasy Mini Kit (Qiagen, Valencia, CA, USA) from mouse iPSC-derived EBs on days 6, 12, 16, and 20; cDNA was synthesized using a RevetAid™ First Strand cDNA Synthesis Kit. The resulting cDNA (100 ng) was amplified by PCR using specific primers. Primer sequences are listed in Table 1.

**Table 1:** Primers for reverse transcription polymerase chain reaction

Gene	Direction	Sequences
Mef2c (NM_025282)	Forward	AGATACCCACAACACACCACGCGCC
	Reverse	ATCCTTCAGAGAGTCGCATGCGCTT
cTnT (NM_001130176)	Forward	CAGAGGAGGCCAACGTAGAAG
	Reverse	TCGATCAGAGTCTGTAGCTCATT
MLC-2 V (NM_010861)	Forward	AAAGAGGCTCCAGGTCCAAT
	Reverse	CCTCTCTGCTTGTGTGGTCA
BLC-2 (NM_009741.3)	Forward	CGATTGTGGCAGTCCCTTA
	Reverse	CCAGGATGAAGTGCTCAGGT
GAPDH (NM_008084)	Forward	TGTGTCCGTCGTGGATCTGA
	Reverse	TTGCTGTTGAAGTCGCAGGAG

The RT-PCR was performed with GAPDH mRNA as a normalizing internal control. Thermal cycling (in 20 μL) was performed as follows: a 3 min denaturation at 95 °C, 30 cycles of 94 °C for 30 s, 59 °C for 30 s and 72 °C for 1 min, and a final extension for 6 min at 72 °C. PCR products were resolved by electrophoresis on 1.5 % agarose gels. They were visualized by UV transillumination and photographed. Semi-quantitative analysis was done by Alphaview 1.3 software (Alpha Lnnotech Inc.).

### Real-time quantitative PCR

Real-time quantitative PCR (qPCR) was performed as described previously [12]. Briefly, the processes of RNA extraction and reverse transcription of RNA were the same to semi-quantitative RT-PCR. The qPCR amplification reactions were performed in a final volume of 20 μL containing 200 ng cDNA, 10 μL of 2× iQSYBR-green mix (Takara, Japan), 300 nmol of each primer using a Bio-Rad CFX96. Thermal cycling was performed as follows: a 5 min denaturation at 95 °C, 40 cycles of 95 °C for 15 s, 59 °C for 30 s and 72 °C for 30 s. Specificity of amplification was determined by DNA melting curve during gradual temperature increments (0.5 °C).

### Confocal microscopy

Cells were fixed in 4 % paraformaldehyde for 30 min, permeabilized for 15 min with 0.25 % Triton X-100, and blocked in 5 % BSA for 15 min. The cells were then incubated with the corresponding primary antibodies for 4 h at room temperature. Primary antibodies (1:200 dilutions) included anti-MLC-2 V, anti-α-actinin, anti-Ki67, anti-connexin 43 (Cx43) (rabbit polyclonal, Abcam) and mouse anti-cardiac troponin T (cTnT) (Abcam). After adequately washed with PBS, cells were incubated at room temperature for 1 h to corresponding FITC-conjugated anti-rabbit or Cy3-conjugated anti-mouse antibodies (Abcam, 1:200 dilution). DAPI (Invitrogen, 1:1,000 dilution) staining was used to identify nuclei. Analysis was performed using a confocal microscope (FV1000, Olympus).

### Western blot

Cellular lysates were prepared as described previously [13]. Proteins were resolved using SDS-PAGE and transferred to immobilon polyvinyldi-fluoride membranes (Millipore, Billerica, MA, US). The membranes were blocked with 4 % BSA for 1 h at roomtemperature and then probed with rabbit antibodies against mouse phosphorylated FAK (p-FAK), FAK, phosphorylated JNK (p-JNK), JNK, or β-actin (Cell Signaling Technology, 1:1000) for 1 h at room temperature. After three washes, the blots were incubated with a goat anti-rabbit peroxidase-conjugated secondary antibody (1:3000) for 1 h at room temperature. The blots were visualized with enhanced chemiluminescence and exposed in DOCXRS^+^ (Bio-Rad, American). Then, proteins were quantitatively determined by densitometry using Image Lab V4.1 software.

### Small interfering RNA for FAK

FAK small interfering RNA (FAK siRNA) (Santa Cruz, CA) is target-specific 19–25 nt siRNAs designed to knock down gene expression. iPSCs were transfected with 3 nmol of FAK siRNA as described previously [14] with minor changes. Transfection of siRNA into iPSCs was carried out in a 6-well plate using transfection reagent according to the manufacturer’s protocol (Santa Cruz Biotechnology). The medium was removed 6 h after transfection followed by the addition of 3 ml fresh medium. Control siRNA is a siRNA sequence that will not cause the specific degradation of any cellular message. After siRNA transfection, the p-JNK in the whole cell lysates was determined by western blot assay and the mRNA expression of Mef2c, Bcl-2, cTnT and MLC-2V was determined by a RT-PCR.

### Statistical analysis

The experiment was repeated at least three times. All values are presented as mean ± SEM. Statistical significance for two comparisons was evaluated by the Student’s paired or unpaired *t* test (two-tail). One-way ANOVA followed by Newman Keuls test was used for multiple comparisons. Differences with *p* < 0.05 were considered statistically significant.

## Results

### Myocardial differentiation of iPSCs

**Fig. 1:**
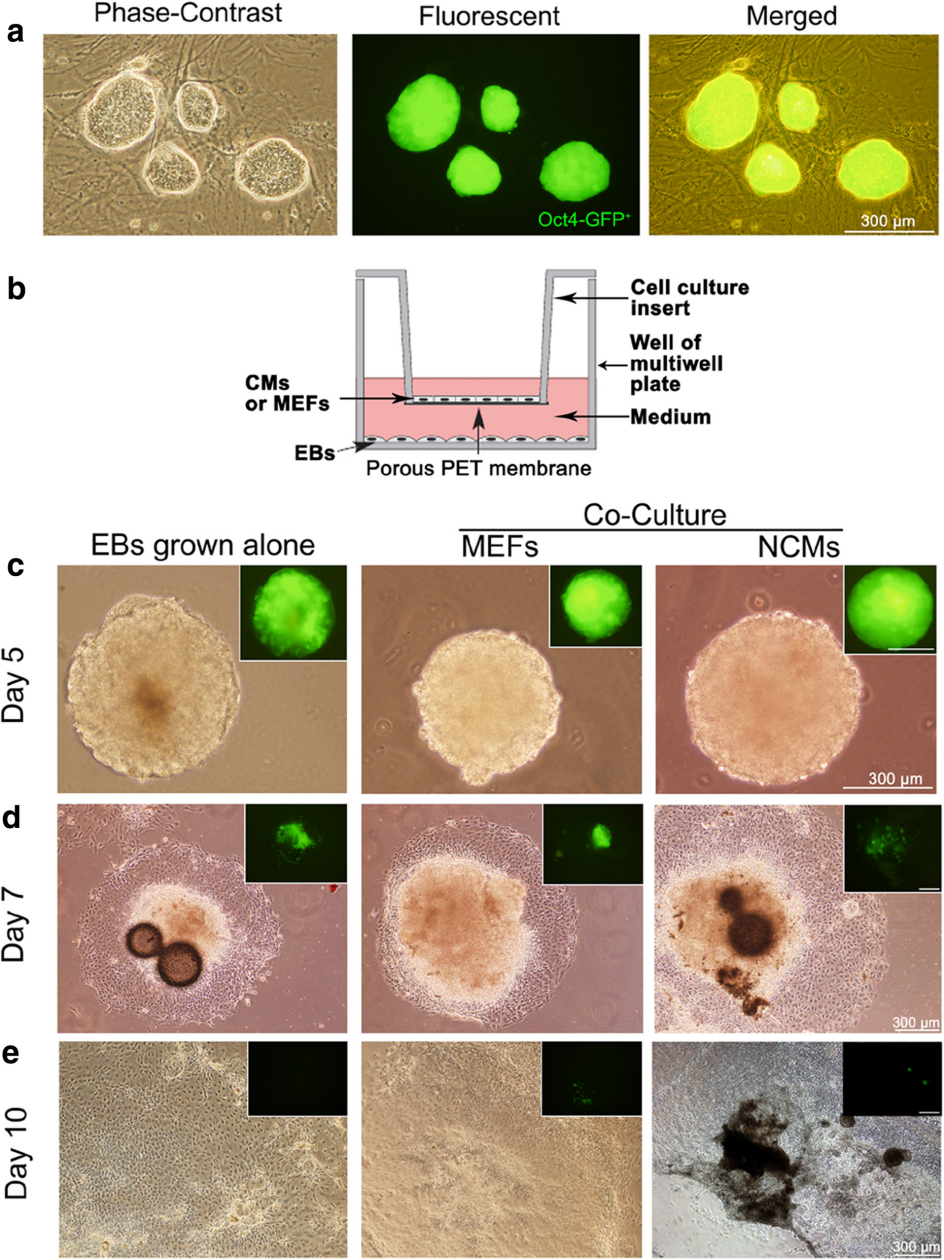
Myocardial differentiation of induced pluripotent stem cells (iPSCs) in the indirect co-culture model. **a** the culture of undifferentiated iPSC colonies with Oct4-GFP^+^ expression (green) on feeder cells. **b** The indirect co-culture system: a facile cell expansion and stem cell differentiation system with continuous medium conditioning while preventing mixing by hanging culture inserts. Two cell populations that are co-cultured in different compartments (insert and well) but can communicate via paracrine signaling through the pores of the membrane. **c**, **d**, **e** show the development of embryoid bodies (EBs) from 5 to 10 days in grown alone, co-culture with mouse embryo fibroblasts (MEFs), and co-culture with neonatal cardiomyocytes (NCMs) (**c**, 5-day-old EB cultured in suspension. **d**, 7-day-old EB. **e**, 10-day-old EB). Hanging inserts removed when photographed). There are few remaining GFP+ colonies (green) after 10 days. Scale bar =300 μm

**Fig. 2:**
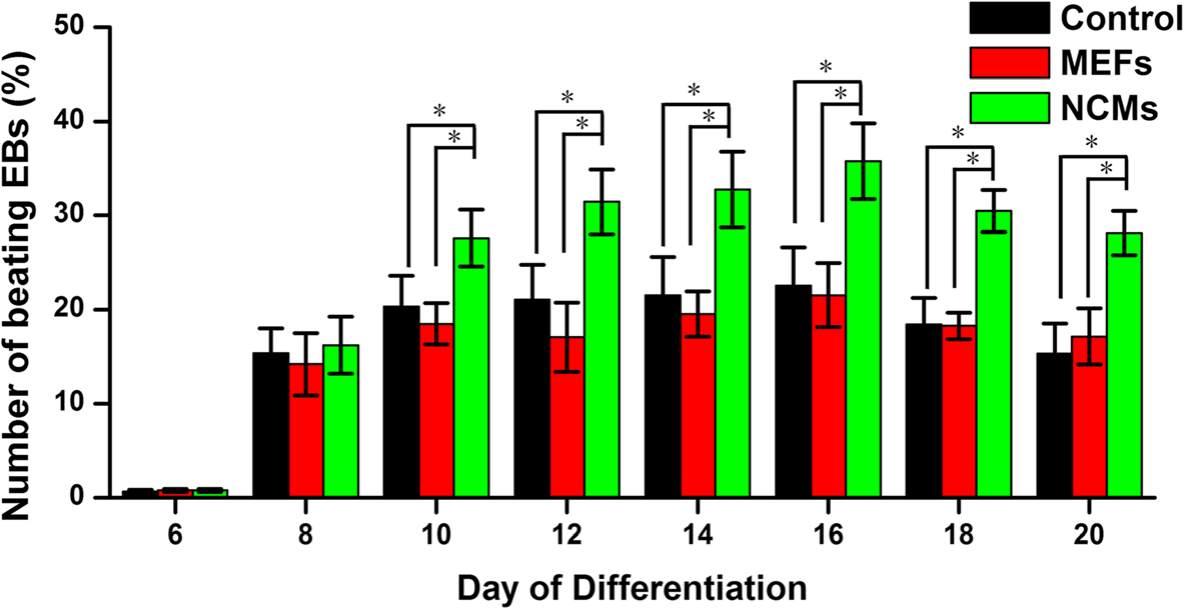
Effect of co-culture on the differentiation efficiency of iPSCs. Time course quantification of spontaneous beating activity of differentiated CMs was expressed as the percentage of beating EBs. *: *P* < 0.05 versus control or MEFs co-culture

iPSCs were cultured with feeder cells in the mentioned iPSC medium. Figure 1a shows undifferentiated iPSC colonies with Oct4-GFP^+^. In vitro myocardial differentiation of iPSCs was induced using a co-culture model established in our laboratory (Fig. 1b). In the co-culture model, the MEFs or NCMs were seeded on 12- well hanging cell culture inserts to prevent direct contact with the subnatant EBs. During the differentiation process, serial phenotypic changes of EBs were analyzed (Fig. 1c, d, e). Initially, these bodies were formed by hanging drop culture and largely composed of densely packed iPSCs, creating simple EBs. After cultured in suspension 2–3 days, the EBs adhered to plates and the center of the bodies became cavitated. The oct4-GFP^+^ colonies began to decrease, even to disappear, during differentiation process. From day 8 onwards, spontaneously contracting cells appeared as clusters in outgrowths from the EBs (see Additional files 1 and 2), suggesting the occurrence of functional CMs in EBs, that it is iPS-CMs. The properties of iPS-CMs may be relatively immature, that the phenotype of CMs within the contracting EBs, comprised of atrium-like, ventricle-like, and node-like cells, may be diverse [15]. More percentage of beating EBs were observed in NCM co-culture (Fig. 2). However, the percentage of beating EBs in MEFs co-culture was similar to the EBs grown alone. So we further study the myocardial differentiation in NCM co-culture, while EBs grown alone or MEF co-culture were used as a control.

### Co-culture increased the proliferation of iPS-CMs

**Fig. 3:**
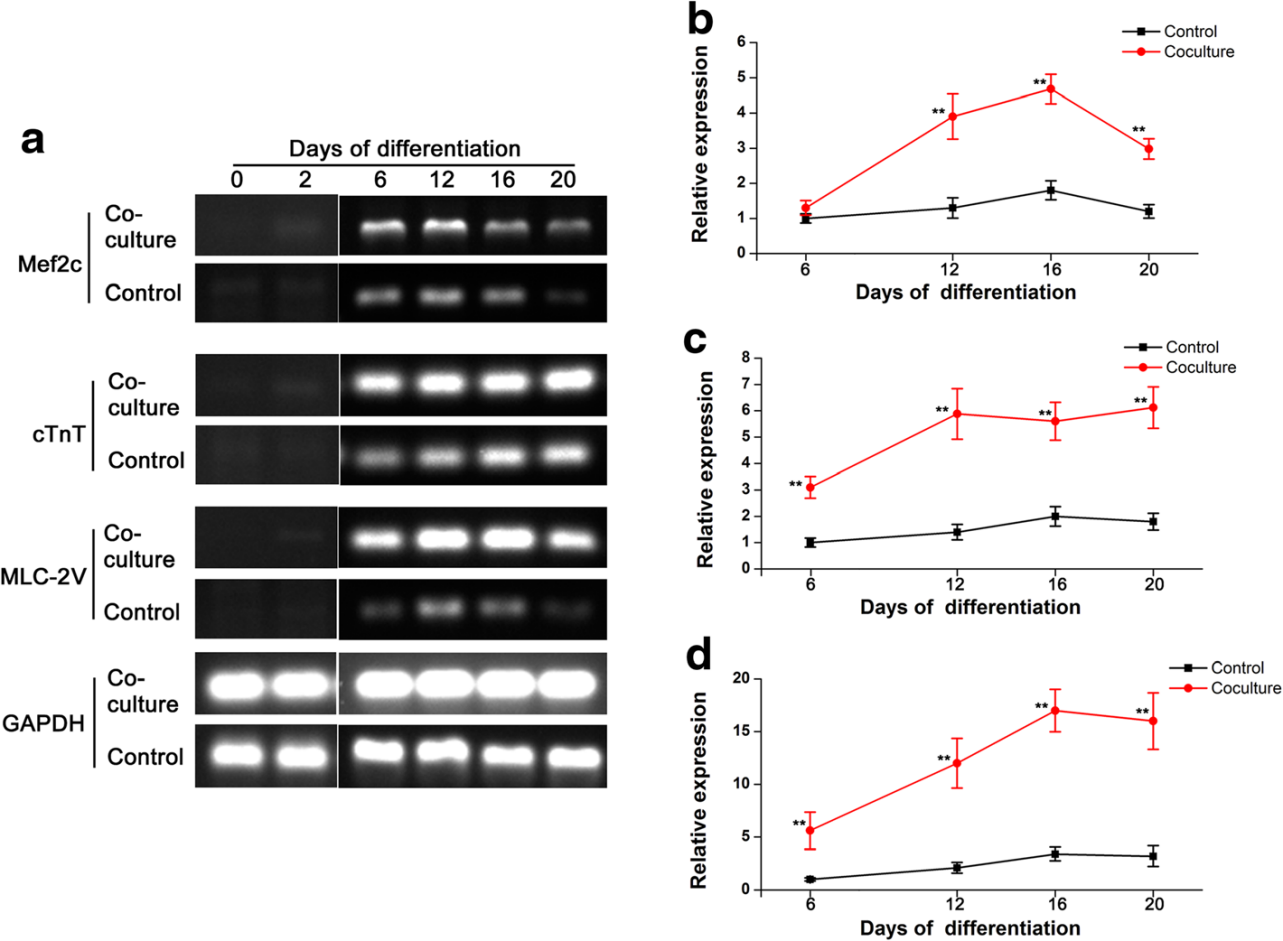
The effects of co-culture on the myocardial differentiation. Reverse Transcription-PCR analysis of cardiac marker genes (Mef2c, Cardiac Troponin T, and MLC-2 V) during the myocardial differentiation of induced pluripotent stem cells (iPSCs) (days 6–20). Glyceraldehyde-3-phosphate dehydrogenase (GAPDH) was used as a normalizing internal control. **a**, the results of Semi-quantitative RT-PCR. **b**, **c**, **d** show the quantitative analysis of cardiac marker gene expression: relative expression over the control at day 6. (**b**, Mef2c. **c**, Cardiac Troponin T. **d**, MLC-2 V). **: *p* < 0.01 versus control

RT-PCR showed high expression levels of Mef2c, cTnT, and MLC-2V in the co-culture on days 6, 12, 16, and 20 (Fig. 3a). Real-Time PCR revealed that the Mef2c, cTnT, and MLC-2V have increased expressions during the differentiation process in the co-culture. Compared with the control, Mef2c expression (Fig. 3b) was significantly enhanced on days 12, 16 and 20, while cTnT expression (Fig. 3c) and MLC-2V expression (Fig. 3d) were gradually increased during the differentiation process. However, late-term cultured cells had a rate of apoptosis that results in a decreased gene expression after day 20.

**Fig. 4:**
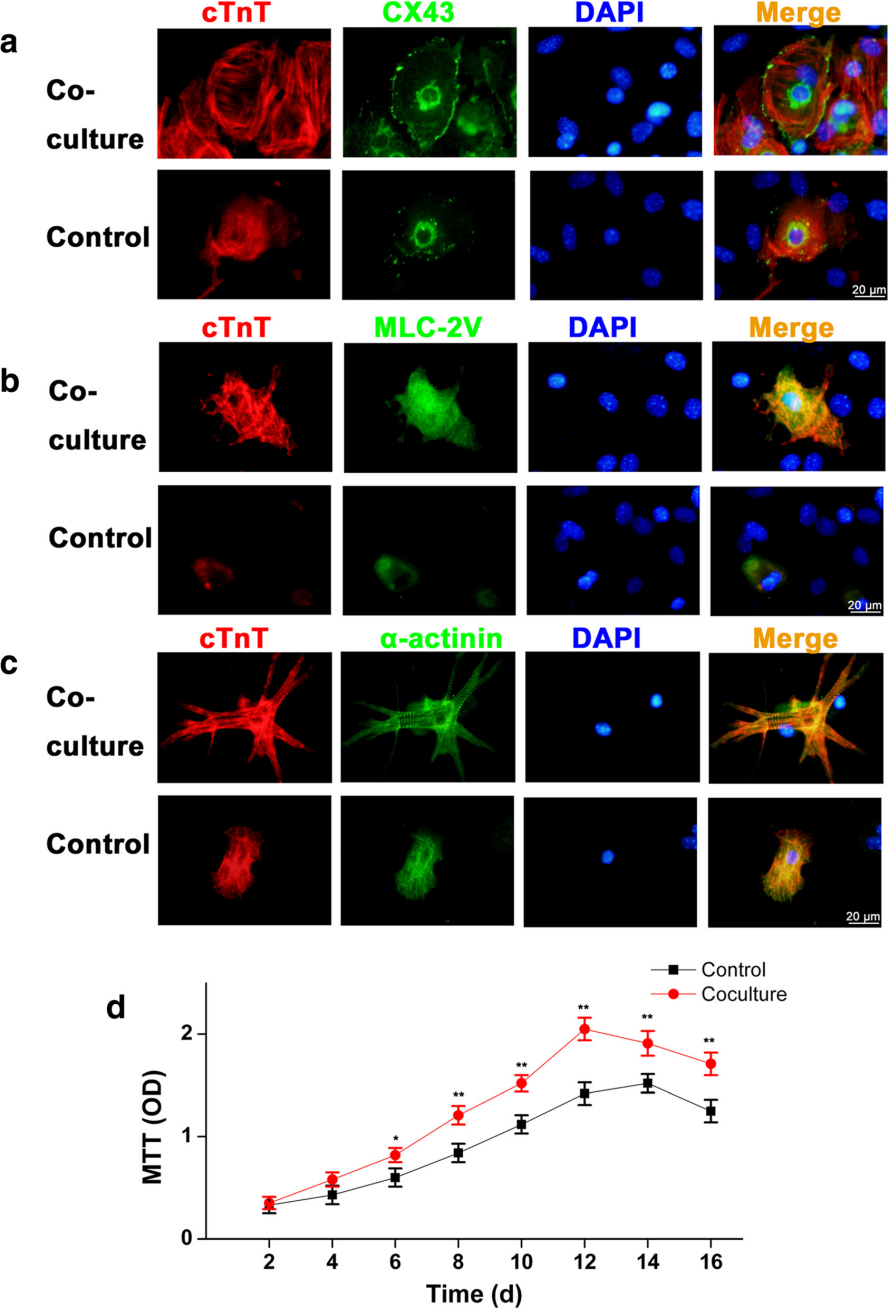
Expression of cardiac specific proteins in induced pluripotent stem cell-derived cardiomyocytes (iPS-CMs) on day 12. **a**, double immunostaining of cells by antibodies against cTnT (red) and CX43 (green). **b**, double immunofluorescent staining for cTnT (red) and MLC-2 V (green), **c**, double immunofluorescent staining for cTnT (red) and α-actinin (green). Nuclei in the same field were stained with DAPI (blue). Merged figures were made by FV10-ASW Systems. **d**, growth curves of cells in co-culture and control analyzed by a MTT assay. The growth of the cells in co-culture was significantly better than that in control after day 6. Scale bar =20 μm,*: *P* < 0.05 versus control, **: *P* < 0.01 versus control

Immunohistochemical analysis of iPS-CMs showed that the expression and distribution of the cardiac-specifc proteins cTnT, MLC-2 V, and α-actinin, was more obvious than that in the control. In addition, connexin 43 (Cx43) was expressed at cell-to-cell contacts in cardiac clusters, and Cx43 staining indicated the presence of gap junctions between cells in cardiac clusters. More mature sarcomere can be found in the co-culture (Fig. 4a, b, c). In order to check whether co-culture could affect the proliferation of EBs, MTT assay was performed. The growth of the EBs in co-culture was significantly better than that in control after day 6 (Fig. 4d).

### The signaling pathways of FAK and JNK are involved in the differentiation of iPSCs

**Fig. 5:**
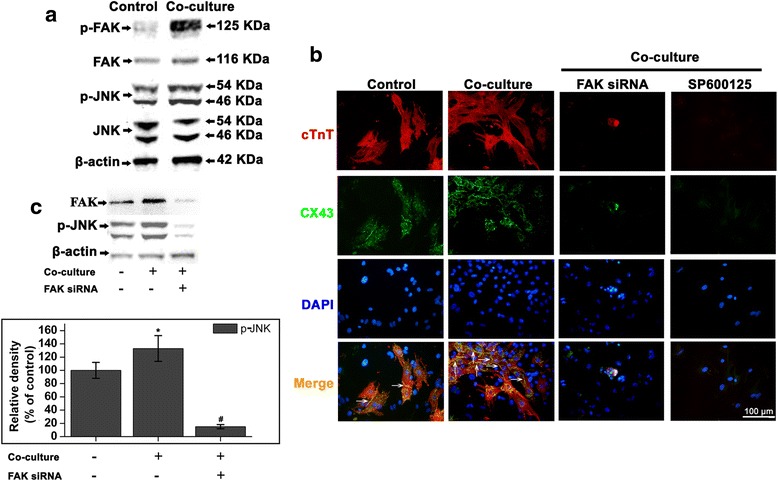
Focal adhesion kinase (FAK) and c-Jun N-terminal kinase (JNK) are involved in coculture-mediated myocardial differentiation. **a**, the phosphorylation of FAK and JNK were performed. **b**, cells were transfected with FAK siRNA or treated with specific JNK inhibitor (SP600125), the double immunostainings for cTnT (red) and CX43 (green) was examined, nuclei in the same field were stained with DAPI (blue). Note the presence of gap-junctions (immunostaining for Cx43, green) at the interphase (arrow heads) between the cells. **c**, West blot showed that the cells transfected with FAK siRNA expressed lower levels of FAK protein than controls. In addition, the p-JNK protein level was also significant decreased after transfected with FAK siRNA, compared to the control at the same time. Scale bar =100 μm, *: *P* < 0.05 versus control, #: *P* < 0.01 versus control

To investigate whether the enhanced CM generation of iPSCs was related to the phosphorylation of FAK, we performed western blot to examine the p-FAK on day 12. In the co-culture, phosphorylation of FAK at tyrosine 397 sites (p-FAK Tyr397) was increased signifcantly. To further study the mechanisms, the phosphorylation of JNK were examined and we found that the p-JNK was also upregulated in iPS-CMs (Fig. 5a).

Furthermore, the iPSCs were transfected with FAK siRNA (Santa Cruz Biotechnology), or treated with specific JNK inhibitor (SP600125, Selleck, USA, 20 μM). The double immunostainings for cTnT and CX43 indicates that the differentiation of iPSCs were attenuated by FAK siRNA or SP600125 (Fig. 5b). Cells transfected with FAK siRNA expressed lower levels of FAK protein than controls. In addition, the p-JNK protein level was also reduced dramatically by FAK siRNA, by 87.4 % (Fig. 5c). These results indicate the successful transfection of FAK siRNA into iPSCs targeting down-regulation of p-JNK protein.

### CM proliferation were reduced by JNK inhibitors

**Fig. 6:**
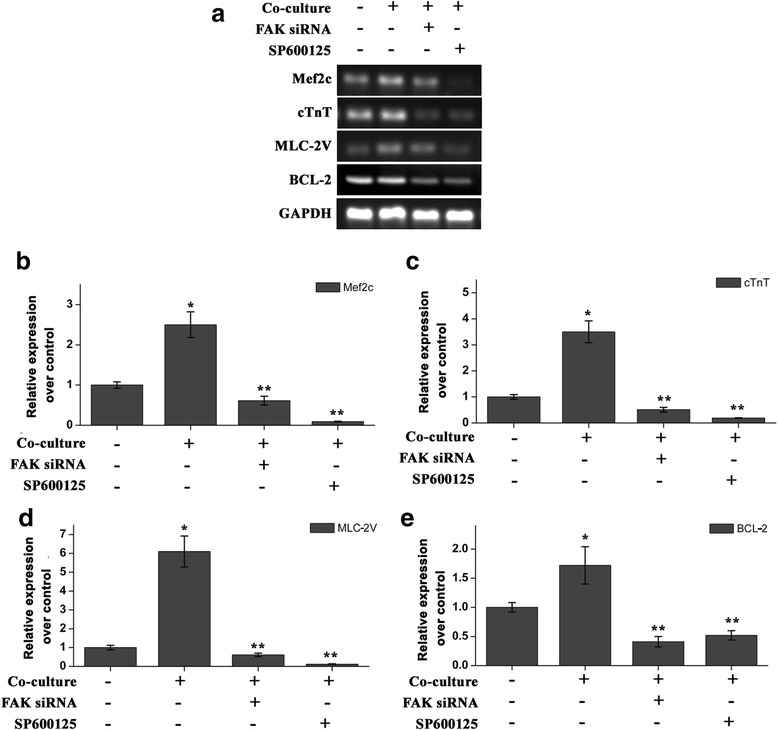
The effects of focal adhesion kinase (FAK) siRNA or SP600125 on myocardial differentiation. **a**, the expression of Mef2c, cTnT, MLC-2 V and BCL-2 after pretreation of FAK siRNA or SP600125 was examined by RT-PCR. **b**, **c**, **d**, **e**, the quantitative analysis on Mef2c, cTnT, MLC-2V, and Bcl-2 expressions after pretreation of FAK siRNA or SP600125 by a real-time PCR. *: *P* < 0.01 versus control, **: *P* < 0.01 versus co-culture

We next used a RT-PCR assay to investigate the inhibitory effect of FAK or JNK on the mRNA levels of Mef2c, cTnT, MLC-2 V and BCL-2, to further confirm FAK/JNK pathways were involved in myocardial differentiation. The expression of cardiac-specifc molecules such as Mef2c, cTnT, and MLC-2 V was decrease by inhibitory of FAK or JNK (Fig. 6a, b, c, d). In addition, the Bcl-2 expression were also significantly decreased after treated with FAK siRNA or SP600125 (Fig. 6e). These results reveal that JNK might have an effect on the myocardial differentiation.

**Fig. 7:**
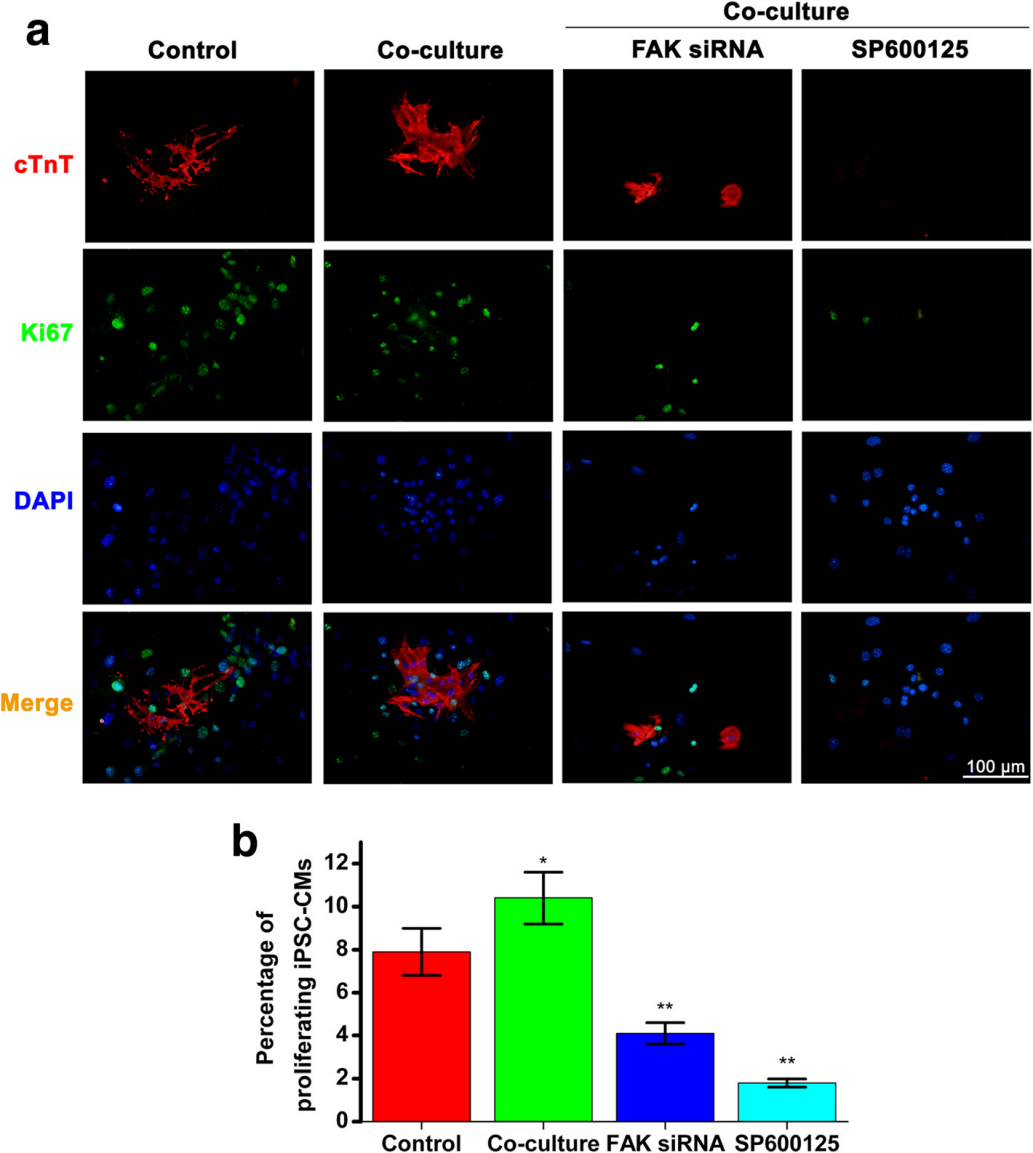
The proliferative capacity of the induced pluripotent stem cell-derived cardiomyocytes (iPS-CMs). **a**, double immunostainings for Ki67 (a marker for cycling cells, green) and cTnT (a marker for CM, red), nuclei in the same field were stained with DAPI (blue). **b**, the percentage of proliferating iPS-CMs in control (7.9 ± 1.1 %), co-culture (10.4 ± 1.2 %), FAK siRNA (4.1 ± 0.5 %), and SP600125 (1.8 ± 0.2 %). Scale bar =100 μm, *: *P* < 0.01 versus control, **: *P* < 0.01 versus co-culture

An alternative explanation to the higher number of iPS-CMs in the co-culture compared with those EBs grown alone may be related to alteration of the proliferative capacity of the iPS-CMs. To quantify this aspect, we performed double immunostainings for Ki67 (a marker for cycling cells) together with cTnT (Fig. 7a). The percentage of proliferating iPS-CMs in the co-culture (10.4 ± 1.2 %) was higher than those of the control (7.9 ± 1.1 %) (*P* < 0.05), while the FAK siRNA resulted in 4.1 ± 0.5 % proliferating iPS-CMs (*P* < 0.01) and SP600125 resulted in 1.8 ± 0.2 % proliferating iPS-CMs (*P* < 0.01) (Fig. 7b).

## Discussion

Previously, many reports suggest that co-cultured stem cells with other cell line results in significantly altered phenotype and differentiated characteristics [16–18]. Here, co-culture of EBs and NCMs was established in a transwell insert system, while EBs grown alone as controls. We used the co-culture model instead of conditioned medium to treat the EBs and found that iPSCs can efficiently differentiate into CMs. The expression levels of Mef2c, cTnT, and MLC-2V were higher in the co-culture during differentiation process. Mef2c is a transcription factor of cardiac myogenesis and right ventricular development [19]. cTnT and MLC-2V are cardiac specific structural proteins. As described previously [20], cTnT, MLC-2V andα-cardiac actin are the markers of CM maturation during the differentiation of embryonic stem cells into CMs. The increased expression levels of Mef2c, cTnT, and MLC-2V indicate an efficient myocardial differentiation in co-culture. The cardiac-specifc proteins, such as cTnT, MLC-2V, α-actinin, and CX43, were more obvious than that in the control. In addition, the growth of the cells in co-culture was significantly better than that in control after day 6. These results indicate NCM co-culture increased the differentiation and proliferation of iPS-CMs.

Paracrine factors released from the cells should play a major role in cell communication on the signalling processes for cell growth and development. In vivo, transplantation of stem cells into heart resulted in cardiac differentiation. In infarcted myocardium, grafted stem cells differentiated into functional CMs integrated with surrounding tissue, improving contractile performance. The transplanted stem cells are directed to differentiate into CMs by signaling mediated through a cardiac paracrine pathway. In vitro, the NCMs may provide a cardiac paracrine pathway that induced a cardiac phenotype in stem cells that just like in vivo. The medium in co-culture may contain more cytokines and growth factors e.g. tissue inhibitor of metalloproteinase-1, vascular endothelial growth factor, macrophage inhibitory factor, fibronectin and connexin 40 [21, 22]. Mechanistically, the paracrine factors may active FAK/JNK signaling in EBs showed a higher level of FAK-phosphorylation. The interaction between iPSCs and NCMs led to a modified gene expression and induction of proliferation.

FAK is an integrin-associated protein tyrosine kinase that is important in cell-cell interactions, which plays a central role in the regulation of cell proliferation, survival, differentiation, and migration [5]. Previously, we found that FAK expression is induced concurrently with the emergence of CM progenitor cells during differentiation of iPSCs, which were consistent with the previous report that FAK plays an essential role in chamber outgrowth and looping morphogenesis during multi-chambered heart formation [23, 24]. In the co-culture with NCMs, phosphorylation of FAK at tyrosine 397 sites (p-FAK Tyr397) was increased signifcantly on day 12 in the differention process. JNK plays a critical role in the induction of gene transcription [8] and has been reported to be the downstream effector of FAK [25]. We examined the potential role of JNK in the signaling pathway that is involved in coculture-mediated myocardial differentiation. The iPSCs were transfected with FAK siRNA or treated with specific JNK inhibitor (SP600125) resulted in the double immunostainings for cTnT and CX43 were visibly attenuated. At the same time, p-JNK protein also reduce dramatically. FAK siRNA or SP600125 also reduced the expression levels of Mef2c, cTnT, and MLC-2 V, which resulted in a poor myocardial differentiation. The expression of Bcl-2 indicates cell proliferating [26]. The Bcl-2 expression was significantly decreased after treated with FAK siRNA or SP600125.

FAK can be activated in response to diverse stimuli and plays an important role in the proliferation and metastasis of cells. A variety of extracellular matrix proteins, including collagen type, lead to an increase in tyrosine phosphorylation and activation of FAK [27]. In melanoma cells, the increased expression of FAK correlates with increased cell motility [28]. Also, FAK is at the crossroad of several signaling pathways, including PI3K/Akt and MAPK pathways [29]. Our study showed that extracellular environment in co-culture with specific cell is also an important factor to activate the FAK signaling.

Ki67 is a marker for cells that either are or have recently been in the cell cycle [30]. An alternative explanation to the higher differentiation of iPS-CMs in the co-culture compared with those EBs grown alone may be related to alteration of the proliferative capacity of the iPS-CMs. We performed double immunostainings for Ki67 and cTnT and found that the percentage of proliferating iPS-CMs in the co-culture was higher than those of the control, while the FAK siRNA resulted in 4.1 ± 0.5 % proliferating iPS-CMs and SP600125 resulted in 1.8 ± 0.2 % proliferating iPS-CMs.

## Conclusion

These experimental data demonstrate that co-culture of EBs with NCMs induces genes expressed in a mature pattern and stimulates the proliferation of iPS-CMs by activating FAK/JNK signaling. Different from native CMs, the iPS-CMs have a certain ability to proliferate. NCM co-culture may be an factor to enhance the proliferated ability of iPS-CMs through activating FAK/JNK. It is interesting, but need more research to study the relevant mechanisms.

### Ethics approval and consent to participate

Animal experiments were approved by the Laboratory Animal Ethics Committee of the Fourth Military Medical University and were conducted in accordance with national guidelines for the care and use of laboratory animals.

### Availability of data and materials

The datasets supporting the conclusions of this article are included within the article and its additional files.

## Additional files

Additional file 1: Video1. The beating outgrowths of embryoid bodies (EBs) from induced pluripotent stem cells (iPSCs) on day 8. (AVI 446 kb) Additional file 2: Video2. The beating outgrowths of embryoid bodies (EBs) from induced pluripotent stem cells (iPSCs) on day 12. (AVI 472 kb)

## Abbreviations

CMs: cardiomyocytes
cTnT: cardiac troponin T
EBs: embryoid bodies
ESC: embryonic stem cell
FAK: focal adhesion kinase
iPS-CMs: iPSC-derived cardiomyocytes
iPSCs: induced pluripotent stem cells
JNK: c-Jun N-terminal kinase
Mef2c: myocyte-specific enhancer factor 2C
MLC-2V: myosin light chain-2V
NCMs: neonatal cardiomyocytes

## References

[1] Zwi L, Caspi O, Arbel G, Huber I, Gepstein A, Park IH, Gepstein L (2009). Cardiomyocyte differentiation of human induced pluripotent stem cells. Circulation.

[2] So KH, Han YJ, Park HY, Kim JG, Sung DJ, Bae YM, Yang BC, Park SB, Chang SK, Kim EY (2011). Generation of functional cardiomyocytes from mouse induced pluripotent stem cells. Int J Cardiol.

[3] Ma J, Guo L, Fiene SJ, Anson BD, Thomson JA, Kamp TJ, Kolaja KL, Swanson BJ, January CT (2011). High purity human-induced pluripotent stem cell-derived cardiomyocytes: electrophysiological properties of action potentials and ionic currents. Am J Physiol Heart Circ Physiol.

[4] Ou DB, Zeng D, Jin Y, Liu XT, Teng JW, Guo WG, Wang HT, Su FF, He Y, Zheng QS (2013). The long-term differentiation of embryonic stem cells into cardiomyocytes: an indirect co-culture model. PLoS One.

[5] Mitra SK, Hanson DA, Schlaepfer DD (2005). Focal adhesion kinase: in command and control of cell motility. Nat Rev Mol Cell Biol.

[6] Cox BD, Natarajan M, Stettner MR, Gladson CL (2006). New concepts regarding focal adhesion kinase promotion of cell migration and proliferation. J Cell Biochem.

[7] Hakuno D, Takahashi T, Lammerding J, Lee RT (2005). Focal adhesion kinase signaling regulates cardiogenesis of embryonic stem cells. J Biol Chem.

[8] Vallerie SN, Hotamisligil GS (2010). The role of JNK proteins in metabolism. Sci Transl Med.

[9] Xu P, Davis RJ (2010). c-Jun NH2-terminal kinase is required for lineage-specific differentiation but not stem cell self-renewal. Mol Cell Biol.

[10] Esteban MA, Wang T, Qin B, Yang J, Qin D, Cai J, Li W, Weng Z, Chen J, Ni S (2010). Vitamin C enhances the generation of mouse and human induced pluripotent stem cells. Cell Stem Cell.

[11] Zeng D, Ou DB, Wei T, Ding L, Liu XT, Hu XL, Li X, Zheng QS (2013). Collagen/beta1 integrin interaction is required for embryoid body formation during cardiogenesis from murine induced pluripotent stem cells. BMC Cell Biol.

[12] Moon SH, Ban K, Kim C, Kim SS, Byun J, Song MK, Park IH, Yu SP, Yoon YS (2013). Development of a novel two-dimensional directed differentiation system for generation of cardiomyocytes from human pluripotent stem cells. Int J Cardiol.

[13] Bhushan S, Kondo K, Polhemus DJ, Otsuka H, Nicholson CK, Tao YX, Huang H, Georgiopoulou VV, Murohara T, Calvert JW (2014). Nitrite therapy improves left ventricular function during heart failure via restoration of nitric oxide-mediated cytoprotective signaling. Circ Res.

[14] Lin SH, Shih YW (2014). Antitumor effects of the flavone chalcone: inhibition of invasion and migration through the FAK/JNK signaling pathway in human gastric adenocarcinoma AGS cells. Mol Cell Biochem.

[15] Zhang J, Wilson GF, Soerens AG, Koonce CH, Yu J, Palecek SP, Thomson JA, Kamp TJ (2009). Functional cardiomyocytes derived from human induced pluripotent stem cells. Circ Res.

[16] Bigdeli N, Karlsson C, Strehl R, Concaro S, Hyllner J, Lindahl A (2009). Coculture of human embryonic stem cells and human articular chondrocytes results in significantly altered phenotype and improved chondrogenic differentiation. Stem Cells.

[17] Fujiwara M, Yan P, Otsuji TG, Narazaki G, Uosaki H, Fukushima H, Kuwahara K, Harada M, Matsuda H, Matsuoka S (2011). Induction and enhancement of cardiac cell differentiation from mouse and human induced pluripotent stem cells with cyclosporin-A. PLoS One.

[18] Li H, Tang M, Liang H, Li Y, Wang J, Song Y, Zheng Y, Xi J, Zhang J, Hescheler J (2013). Coculture of embryonic ventricular myocytes and mouse embryonic stem cell enhance intercellular signaling by upregulation of connexin43. Cell Physiol Biochem.

[19] Lin Q, Schwarz J, Bucana C, Olson EN (1997). Control of mouse cardiac morphogenesis and myogenesis by transcription factor MEF2C. Science.

[20] Caspi O, Lesman A, Basevitch Y, Gepstein A, Arbel G, Habib IH, Gepstein L, Levenberg S (2007). Tissue engineering of vascularized cardiac muscle from human embryonic stem cells. Circ Res.

[21] Rosenberg M, Lutz M, Kuhl C, Will R, Eckstein V, Krebs J, Katus HA, Frey N (2012). Coculture with hematopoietic stem cells protects cardiomyocytes against apoptosis via paracrine activation of AKT. J Transl Med.

[22] Zhang Y, Wang D, Cao K, Chen M, Yang X, Tao Y (2014). Rat induced pluripotent stem cells protect H9C2 cells from cellular senescence via a paracrine mechanism. Cardiology.

[23] Vallejo-Illarramendi A, Zang K, Reichardt LF (2009). Focal adhesion kinase is required for neural crest cell morphogenesis during mouse cardiovascular development. J Clin Invest.

[24] Doherty JT, Conlon FL, Mack CP, Taylor JM (2010). Focal adhesion kinase is essential for cardiac looping and multichamber heart formation. Genesis.

[25] Li JP, Fu YN, Chen YR, Tan TH (2010). JNK pathway-associated phosphatase dephosphorylates focal adhesion kinase and suppresses cell migration. J Biol Chem.

[26] Limana F, Urbanek K, Chimenti S, Quaini F, Leri A, Kajstura J, Nadal-Ginard B, Izumo S, Anversa P (2002). bcl-2 overexpression promotes myocyte proliferation. Proc Natl Acad Sci U S A.

[27] Mukhopadhyay NK, Gordon GJ, Chen CJ, Bueno R, Sugarbaker DJ, Jaklitsch MT (2005). Activation of focal adhesion kinase in human lung cancer cells involves multiple and potentially parallel signaling events. J Cell Mol Med.

[28] Akasaka T, van Leeuwen RL, Yoshinaga IG, Mihm MC, Byers HR (1995). Focal adhesion kinase (p125FAK) expression correlates with motility of human melanoma cell lines. J Invest Dermatol.

[29] Huang C, Jacobson K, Schaller MD (2004). MAP kinases and cell migration. J Cell Sci.

[30] Scholzen T, Gerdes J (2000). The Ki-67 protein: from the known and the unknown. J Cell Physiol.

